# Comprehensive genomic characterization of the soybean *G3PDH* gene family and its role in virus resistance

**DOI:** 10.1186/s12870-025-06579-7

**Published:** 2025-09-01

**Authors:** Kai Huang, Ken-taro Sekine, Shuxin Li, Hao Xu, Daiqiao Song, Pradeep Kachroo, Aardra Kachroo, Hexiang Luan

**Affiliations:** 1 Institute of Plant Genetic Engineering, College of Life Sciences, Qingdao Agricultural University, Qingdao, Shandong 266109 P.R. China; 2 Department of Plant Pathology, University of Kentucky, Lexington, KY 40546 USA; 3 College of Food Science and Engineering, Qingdao Agricultural University, Qingdao, Shandong 266109 P.R. China

**Keywords:** Soybean, G3PDH, Bioinformation analysis, Virus infection, SMV-G7

## Abstract

**Background:**

Glycerol 3-phosphate dehydrogenase (G3PDH) mediated the reduction of dihydroxyacetone phosphate to generate Glycerol 3-phosphate (G3P). G3P plays a significant role in plant anti-viral systemic acquired resistance (SAR). Therefore, it is crucial to systematically characterize the G3PDH gene family, especially its role in virus infection in soybean, to facilitate the cultivation of disease-resistant soybean seeds.

**Results:**

In this study, 10 members of the *G3PDH* family were identified in soybean and renamed according to their chromosomal positions. These members are unevenly distributed across chromosomes. These *G3PDH* genes were divided into five groups through analysis of conserved motifs, gene structure, and phylogenetic analysis. Findings revealed that anaerobic induction response elements, known to be involved in plant stress resistance, were the most abundant among the identified cis-acting elements. Expression analysis revealed that *G3PDH8* exhibited predominant expression in seeds and was significantly upregulated following viral infection. In addition, soybean G3PDH silenced which generated based on bean pod mosaic virus (BPMV) plants accumulated a higher viral load compared to control V plants.

**Conclusions:**

This study provides a systematic characterization of the *G3PDH* gene family, including protein functional features, expression patterns and bioinformatic analysis. Results laid a foundation for exploring the function of *G3PDH* gene in soybean antiviral infection and breeding of soybean for SMV resistance.

**Supplementary Information:**

The online version contains supplementary material available at 10.1186/s12870-025-06579-7.

## Introduction

Soybean is one of the main oil and feed crops worldwide [1], and is an important source of plant protein [2]. Improving yield and quality is an important goal in soybean breeding. However, in soybean diseases, especially soybean mosaic virus (SMV) disease, yield losses as high as 90% have been reported in uniformly infected field plots [3], and the rate of brown spots in diseased seeds can reach more than 35% in serious cases [4], which seriously restricts the high and stable yield and quality of soybean.

SMV is a species within the genus Potyvirus, which includes almost a quarter of all known plant RNA viruses that affect agriculturally important plants including *Glycine max* (cultivated soybean) and G. *soja* (wild soybean), and are transmitted by aphids and seeds [5]. Pathogenic differentiation of SMV occurs during the interaction between SMV and the soybean host, and according to the symptom response of SMV in different soybean hosts, the United States and South Korea have divided SMV into seven strain groups (G1 - G7) [6–10].

The reported research findings indicate that glycerol 3-phosphate (G3P) plays a significant positive role in the disease resistance mechanism of soybean [11]. G3P serves as an essential component in energy production reactions, including glycolysis and glycerophospholipid biosynthesis, and contributes to the disease-related physiology of many organisms [12, 13]. In plants, G3P is produced either through glycerol phosphorylation mediated by glycerol kinase (GK) or through the reduction of dihydroxyacetone phosphate (DHAP) mediated by G3P dehydrogenase (G3PDH). Glycerol-3-phosphate dehydrogenase (G3PDH) was first characterized as a cytochrome-reducing dehydrogenase in 1947 [14]. In eukaryotes, multiple isoforms of G3PDH have been identified, with most isoforms demonstrated to act as critical regulators of stress responses [15–17]. G3PDH regulates the production of G3P, participates in plant disease resistance through systemic acquired resistance (SAR) [18–20], and has a positive effect [15, 21]. *G3PDH* gene mutations result in reduced carbon flux through the plastid pathway of lipid bio-synthesis, which leads to the reduction of hexadecyl trienoic acid (16:3) fatty acid [22, 23]. The defective SAR in *G3PDH* mutant plants is related to a deficiency in the fatty acid-lipid biosynthesis pathway [24]. Thus, G3PDH plays an essential role in plant antiviral processes.

Therefore, we analyzed the *G3PDH* gene family and elucidated the function of one of its family members (Glyma.19G053500). The objective of this study was to investigate the structural and functional alterations in genes throughout the evolutionary process, thereby establishing a basis for soybean breeding through molecular biology methods.

## Materials and methods

### Identification of GmG3PDHs

The whole genome sequence files and gene annotation files of soybean, rice, and alfalfa are from the Ensembl Plant Database (https://plants.ensembl.org/). The soybean coding sequence and target gene sequence were extracted using TBtools software [25]. Gene screening local blast (E-value ≤ 1e-5) completed using TBtools, The HMM of G3PDH domains was obtained from PFAM database (http://pfam.xfam.org/). Finally, predicted proteins were considered as GmG3PDH only if they contained NAD-Gly3Pdh C (PF07479) and NAD-Gly3Pdh N (PF01210) conserved domains verified by TBtools.

### Sequence structure and phylogenetic analysis

The physical and chemical properties of the GmG3PDHs, including their molecular weights (k Da) and isoelectric points (pIs), were analyzed using ExPASy (https://web.expasy.org/protparam/). Motif pattern and gene structures were analyzed using TBtools. Conserved motifs of GmG3PDHs were predicted by MEME on line program (http://meme-suite.org/tools/meme). ClustalX2 software and the neighbor-joining method were used to construct a phylogenetic tree of the *G3PDH* genes in soybean, alfalfa, and rice. The tree was visualized using iTOL v6 (https://itol.embl.de/).

### Gene location and expression patterns

The locations of the genes on the chromosomes were drawn using MG2C (http://mg2C.iask.in/mg2C_v2.1/). Collinearity analysis was performed using TBtools. Two thousand base pairs upstream regions from the start codon site of the *G3PDH* gene were extracted using TBtools and supplied to PlantCARE (http://bioinformatics.psb.ugent.be/webtools/plantcare/html) to predict the sequence data for the *G3PDH* cis-element. Tissue expression heatmap data were obtained from the JGI Phytozome database (Phytozome (doe.gov)) and visualized using TBtools.

### Plant growth and virus strains

The *Glycine max* cultivar Rsv1 was resistant to SMV-G7, whereas *Glycine max* cultivar Essex was susceptible. Plants were grown in an aphid-free greenhouse with day and night temperatures of 25 ℃ and 20 ℃, in 65% relative humidity and during a 14 h photoperiod. The soybean varieties and SMV isolates were maintained in greenhouse, University of Kentucky, United State. All the soybean and viruses were kept in Aardra Kachroo’s lab at the Univeristy of Kentucky.

The SMV isolate G7 was used in this study. The inoculum was prepared by grinding infected Essex leaves in 0.01 mol/L sodium phosphate buffer (3–5 mL/g leaf tissue, pH 7.4) using a mortar and pestle and kept on ice until the inoculation was completed. For the inoculation process, a brush was dipped into the prepared inoculum and then quickly rubbed against the fully expanded opposite cotyledonary leaves of soybean plants. This rubbing action created minute wounds on the leaves, which ensured the successful infection of the virus. The leaves used for inoculation were rinsed with tap water after inoculation. All experiments were carried out under the identical greenhouse conditions, with extraneous variables being carefully controlled to guarantee the consistency of the experimental results.

### Construction of silent materials

The gene silencing experiments involved generation of RNA interference vectors, in vitro transcription of silencing constructs, mechanical inoculation of soybean foliage with recombinant BPMV (Bean pod mottle virus) particles through leaf abrasion were described before [26]. Briefly, a 135 bp fragment (S40-S174) of Glyma19G053500 was used to generate vectors BPMV-RNA2-GmG3PDH which targeting GmG3PDH8. The BPMV-RNA1 and BPMV-RNA2-GmG3PDH were both transcribed in vitro separately and mixed together for rub-inoculation of recombinant BPMV on soybean leaves. Empty vectors were used for the control treatment.

During the polymerase chain reaction, the forward primer was designated as F - AGTGTGGCACAGAACAACTC, while the reverse primer was R - GCTAAGCCTGAGGGTATTGG. The melting temperature of these primers was optimized to 60 °C. The In-Fusion Snap Assembly Master Mix was selected as the enzymatic reagent for the reaction, and the amplification was performed over a total of 30 cycles. For the in vitro transcription step, the HiScribe^®^ T7 High Yield RNA Synthesis Kit was utilized to generate the desired RNA products.

### Detection of viruses in silenced materials

On the indicated days after SMV - G7 infection, V and S_G3PDH_ plants were collected from inoculated leaves and systemic leaves for Western blot detection. Total protein was extracted from leaf tissue using GTEN buffer (50 mM Tris HCl (pH 7.5), 10% glycol, 150 mM NaCl, 10 mM MgCl_2_, 5 mM EDTA, 5 mM DTT, and protein inhibitors). Centrifuge the crude extract at 12,000 rpm for 10 min at 4 ℃ and transfer the supernatant in a sterile tube. Proteins (10–50 mg) were separated by electrophoresis on a 10% SDS-PAGE gel running at 80–130 V for 2 h and then transferred onto a PVDF membrane. The target protein was detected using a primary antibody against SMV-CP. Ponceau staining was used as the control for protein loading.

## Results

### Identification and physicochemical property analysis of *G3PDH* genes in soybean

To identify the members of the *G3PDH* family in soybean, a Hidden Markov Model (HMM) search with a conserved model as a query was performed and combined with sequence alignment. Ten *GmG3PDHs* were identified from the soybean genome and named *GmG3PDH1* - *GmG3PDH10* according to their chromosomal locations **(**Fig. 3**)**. Detailed information on GmG3PDHs, including gene name, gene ID, protein length, molecular weight, and theoretical isoelectric point (pI) distribution, is listed in Table 1. The 10 GmG3PDHs proteins had diverse molecular lengths and weights; GmG3PDH9 had the lowest molecular weight (39.11 k Da), whereas the highest molecular weight (51.73 k Da) was observed in GmG3PDH10. The pI varied from 5.46 (GmG3PDH5) to 9.11 (GmG3PDH2), where eight members had pI values < 7 and two members had pI values > 7, suggesting that most of these genes encode acidic proteins. Subcellular localization analysis revealed that all members of the GAPD family are localized in the cytoplasm. In addition, basic proteins were mainly concentrated in groups A and D (Table 1).

**Table 1:** Basic information of *G3PDH* family members in soybean

Gene	Gene ID	Chr	Num. of AA	MW (k Da)	pI	Average hydrophilicity value	Aliphatic index	Subcellular localisation
*GmG3PDH1*	Glyma.02G186600	2	458	50.89	5.46	-0.082	101.79	Cytoplasmic
*GmG3PDH2*	Glyma.02G218700	2	432	46.68	9.11	0.016	93.47	Cytoplasmic
*GmG3PDH3*	Glyma.03G133800	3	398	44.4	6.73	-0.074	103.14	Cytoplasmic
*GmG3PDH4*	Glyma.05G114400	5	370	40.66	5.65	-0.03	94.27	Cytoplasmic
*GmG3PDH5*	Glyma.10G107100	10	458	50.82	5.46	-0.061	102.23	Cytoplasmic
*GmG3PDH6*	Glyma.11G148900	11	462	51.4	6.21	-0.054	104.7	Cytoplasmic
*GmG3PDH7*	Glyma.12G011200	12	465	51.67	5.89	-0.047	104.65	Cytoplasmic
*GmG3PDH8*	Glyma.19G053500	19	380	41.67	5.48	-0.025	94.37	Cytoplasmic
*GmG3PDH9*	Glyma.19G079400	19	356	39.11	8.18	-0.044	91.46	Cytoplasmic
*GmG3PDH10*	Glyma.19G136100	19	465	51.73	6.77	-0.066	101.72	Cytoplasmic

### Phylogenetic and structural analysis of the GmG3PDHs

**Fig. 1:**
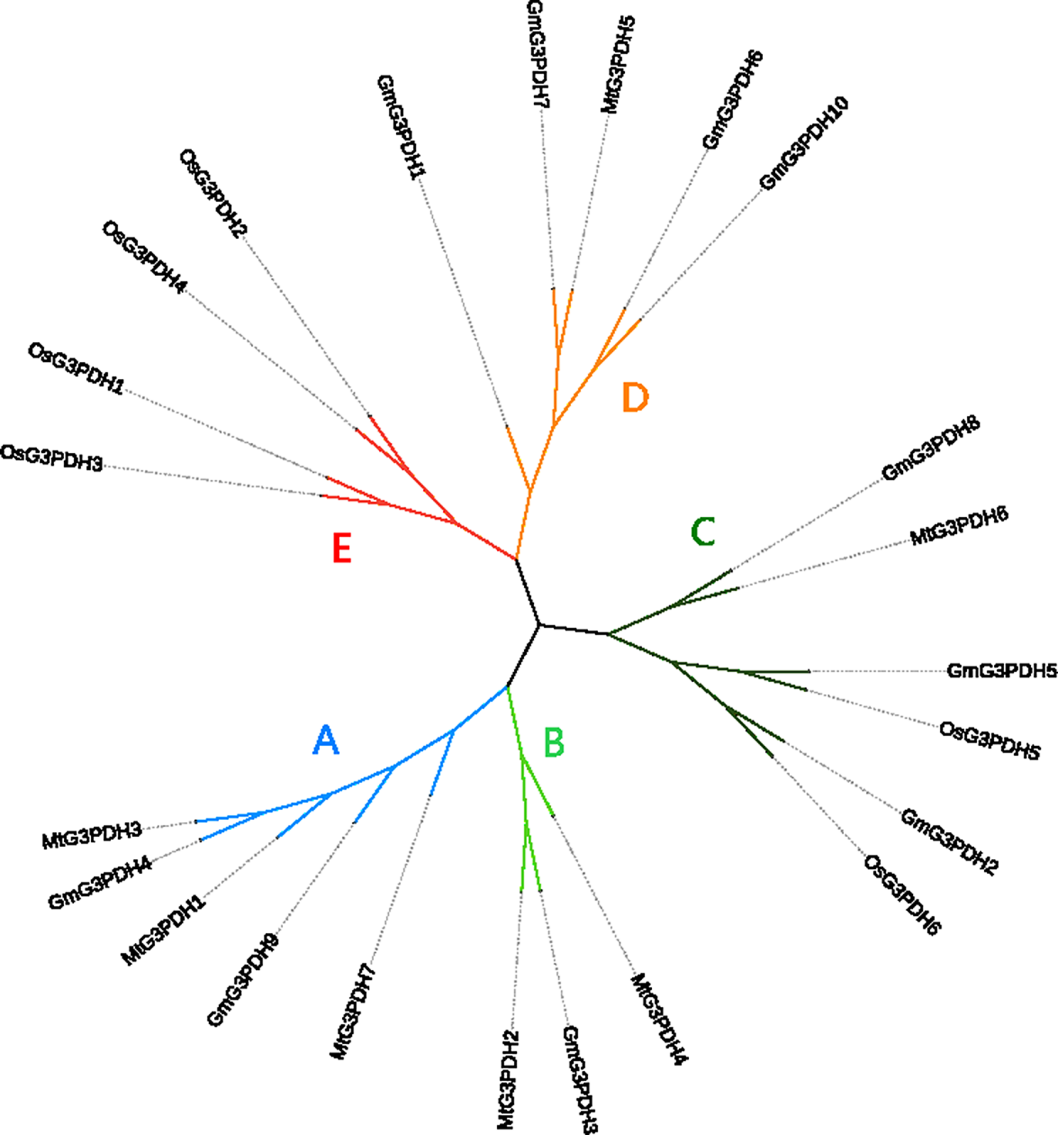
Phylogenetic tree of *G3PDH* genes from three species. These genes were divided into 5 groups and highlighted in different colors

A phylogenetic tree of the *G3PDH* gene family sequences of soybean (*Glycine max*, Gm), alfalfa (*Medicago*, Mt), and rice (*Oryza sativa*, Os) was constructed using ClustalX2 software and the neighbor-joining method to infer the evolutionary relationship of the *G3PDH* gene in different species. Among them, seven genes came from alfalfa and six genes came from rice. *G3PDH* genes were divided into five groups (A-E) based on the results of the phylogenetic tree (Fig. 1). Group A contained three alfalfa *G3PDH* genes and two soybean *G3PDH* genes. Group B contained two alfalfa *G3PDH* genes and one soybean *G3PDH* gene. Group C contained the most members, including two rice *G3PDH* genes, one alfalfa *G3PDH* gene, and three soybean *G3PDH* genes. Group D contained one alfalfa *G3PDH* gene and four soybean *G3PDH* genes. Group E contained the four rice *G3PDH* genes. Notably, the evolutionary relationship of group E was older than that of the others, and all of them were rice *G3PDH* genes. Therefore, we speculate that the evolutionary direction of the *G3PDH* genes in group E in rice has higher specificity. The number of genes in Group C was the highest, encompassing all three species, indicating that the *G3PDH* genes in Group C may have higher environmental adaptability during evolution. It can be seen from the phylogenetic tree that soybean and alfalfa are more closely related in evolution and both belong to dicotyledonous legumes, whereas rice belongs to the monocotyledonous Gramineae.

**Fig. 2:**
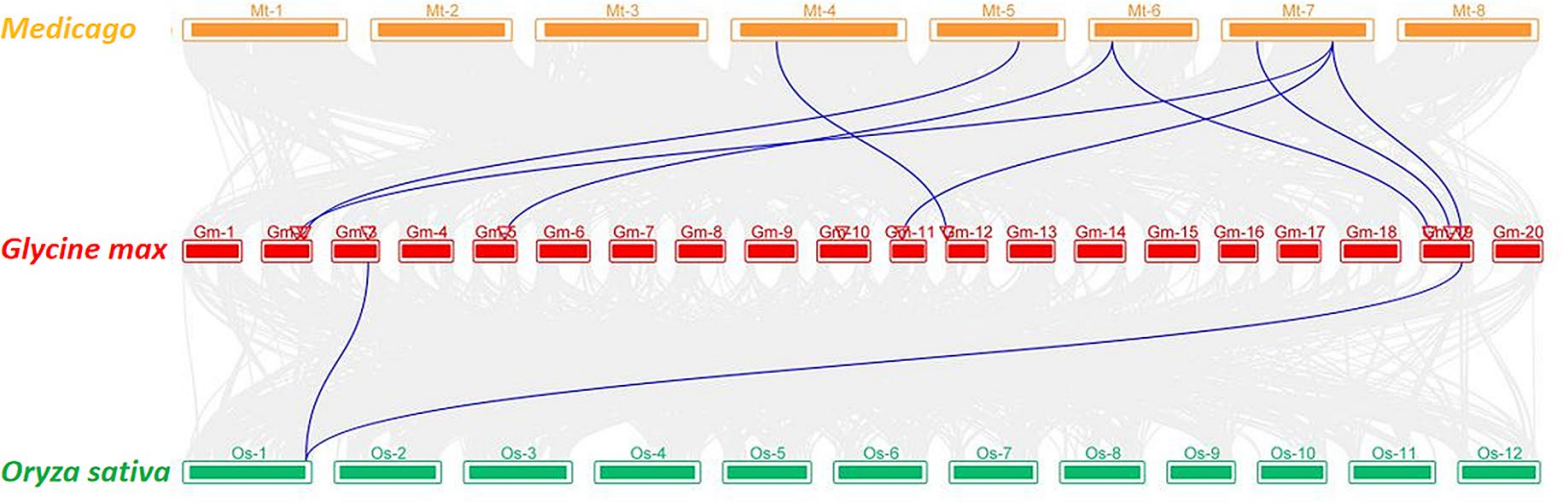
The collinearity analysis of the chromosomes from *Glycine max* (Gm), *Oryza sativa* (Os) *and Medicago truncatula* (Mt). The collinearity blocks include 9 successive homologous genes are connected with blue lines

We performed a collinearity analysis among three species of soybean, rice, and alfalfa (Fig. 2) to better understand the evolutionary mechanism of the *G3PDH* gene family. The results showed that *G3PDH* collinear genes were widely present in alfalfa, whereas only two were present in rice. The number of collinear genes between soybean and alfalfa was higher than that between soybean and rice. Specifically, we observed that *GmG3PDH5* on chromosome 10 lacked homology to rice and alfalfa. Given their shared classification as dicotyledonous leguminous leafy plants, soybean and alfalfa exhibited notably stronger collinearity to rice.

### Distribution and cis-acting elements of *G3PDH* genes on the chromosome of soybean

**Fig. 3:**
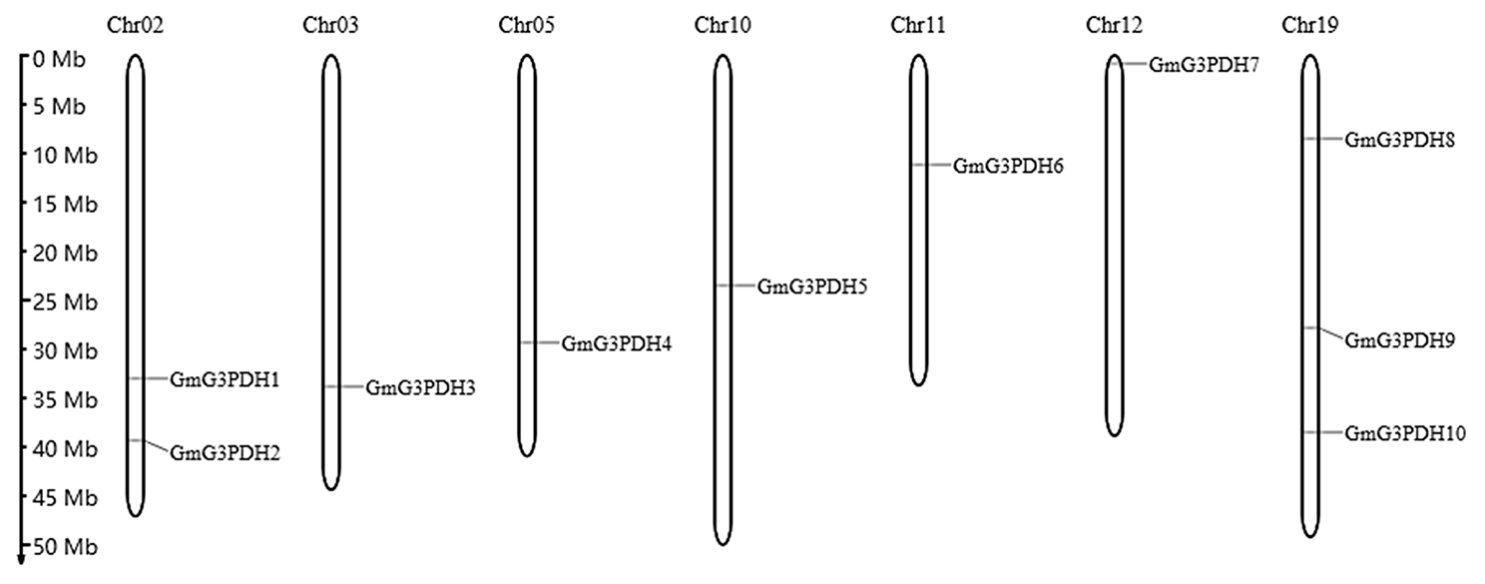
Chromosomal distribution of soybean *G3PDH* genes

In terms of the distribution of genes on the chromosomes (Fig. 3), 10 soybean *GmG3PDHs* were unevenly distributed on seven chromosomes, including two *GmG3PDHs* on Chr02. Chr19 had three *GmG3PDHs* and the largest number of genes, whereas the number of *G3PDH* genes on Chr03, Chr05, Chr10, Chr11, and Chr12 is only one.

**Fig. 4:**
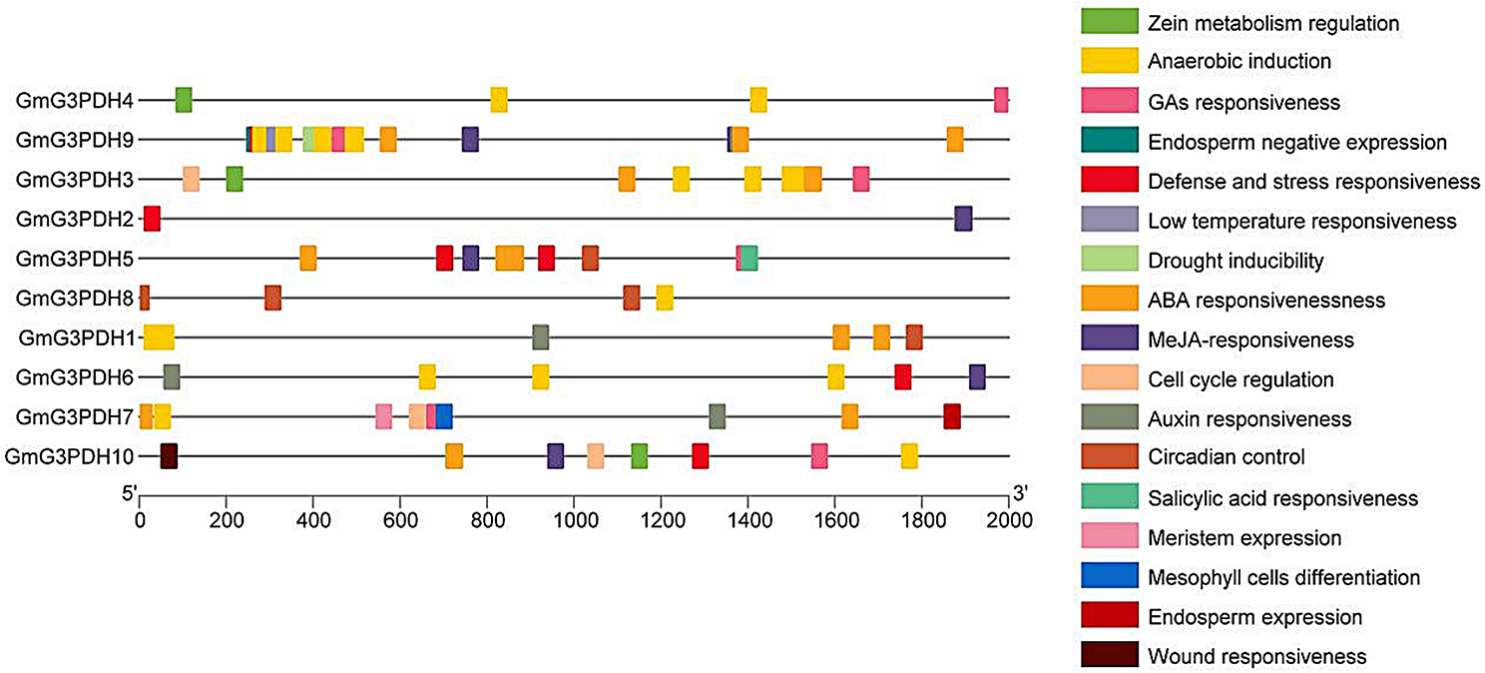
Cis-acting element of soybean *G3PDH* genes

According to the prediction results of promoter elements (Fig. 4), the 10 *GmG3PDHs* exhibit a diverse array of cis-acting elements. These elements include those associated with cell growth and differentiation (such as Endosperm negative expression, Circadian control, Meristem expression, Cell cycle regulation, Mesophyll cells differentiation, Endosperm expression), hormone response (Gibberellins responsive, Abscisic Acid responsive, Methyl jasmonate (MeJA) responsive, zein metabolism regulation, auxin-responsive, salicylic acid-responsive) and stress response (including defense and stress-responsive, anaerobic induction, low temperature responsive, drought inducibility). Among them, anaerobic induction response elements were the most abundant and were detected in all soybean *G3PDH* genes except *GmG3PDH2* and *GmG3PDH5*. Abscisic acid response elements ranked second in number and were present in *G3PDH* genes, except *GmG3PDH2* and *GmG3PDH5*. Overall, these regulatory elements exhibited irregular distribution within the *G3PDH* genes.

### Gene structure and motif composition of the *G3PDH* gene family

**Fig. 5:**
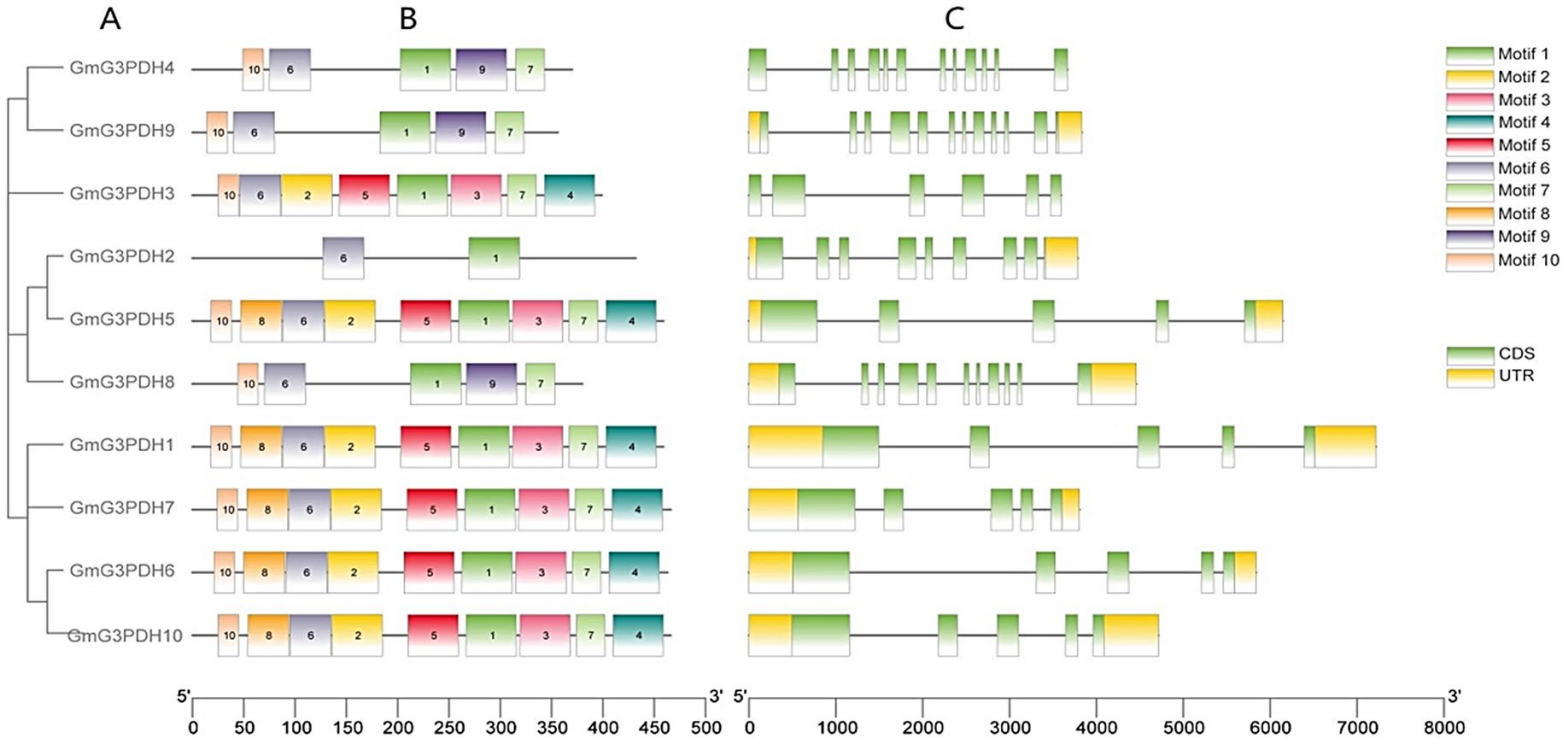
The Phylogenetic tree, motif patten and gene structures of GmG3PDH. (**A**) Construction of an evolutionary tree based on the gene primary structure of GmG3PDHs. (**B**) Conserved Motifs of G3PDH proteins was identified by MEME suite. Each motif is represented by a specific color, with a total of 1–10 motifs. (**C**) Exon-intron organizations of GmG3PDHs. Introns are represented by black lines. Green boxes represent untranslated regions (UTRs) from 5’to 3’, yellow boxes represent coding sequence (CDS)

The conserved motifs in the soybean G3PDH family of proteins were analyzed using the Multiple Expectation Maximization for Motif Elicitation. When the E-value was < 0.05 (significant level), up to 10 motifs were displayed. The conserved motifs (Fig. 5B), gene structure (Fig. 5C), and phylogenetic relationships (Fig. 5A) of G3PDH proteins were visualized using TBtools software.

The structural diversity of exons and introns plays a key role in the evolution of gene families and can provide additional evidence for phylogenetic grouping. The exon-intron structure of the *G3PDH* family members can be divided into two parts: *GmG3PDH1*, *GmG3PDH3*, *GmG3PDH9*, and *GmG3PDH10* are disrupted by more introns (7–10 introns), the coding sequences of *GmG3PDH2*, *GmG3PDH4*, *GmG3PDH5*, *GmG3PDH6*, *GmG3PDH7*, and *GmG3PDH8* are disrupted by fewer introns (3–4 introns). The number of motifs in each G3PDH protein varied from two to nine. GmG3PDH2 only contains motif 1 and motif 6. GmG3PDH4, GmG3PDH8 and GmG3PDH9 have motif 1, motif 6, motif 7, motif 9, motif 10. GmG3PDH1, GmG3PDH5, GmG3PDH6, GmG3PDH7, and GmG3PDH10 contain motifs 1–8 and motif 10. GmG3PDH3 contains motifs 1–7 and motif 10. All family members contain motifs 1 and 6; therefore, we speculate that motifs 1 and 6 were relatively conserved during the evolution of the G3PDH family and play an important role in the function of the G3PDH protein.

### Tissue-specific expression of *G3PDH* genes during soybean development

**Fig. 6:**
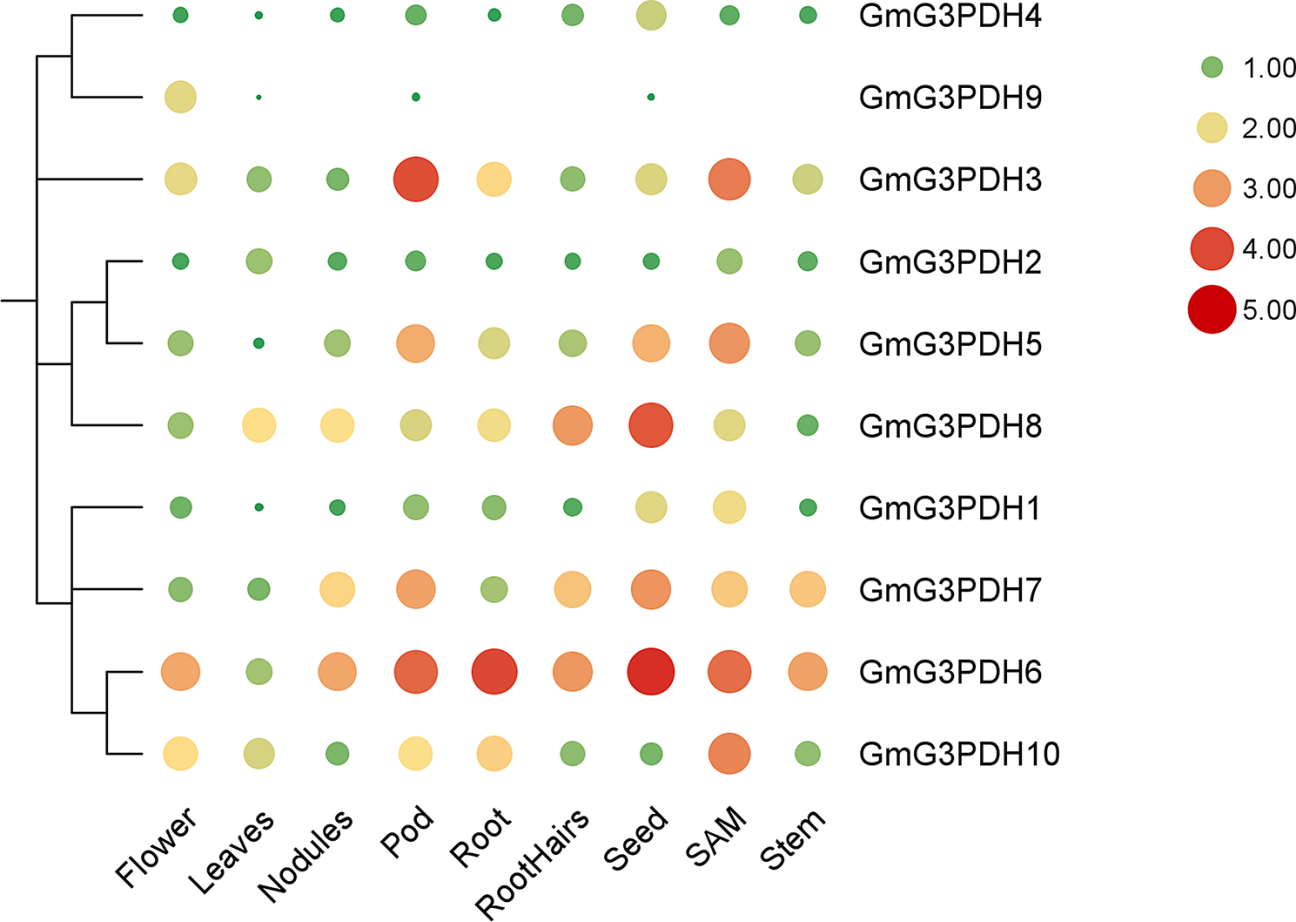
Expression analysis of *GmG3PDHs* during growth and development of different tissues of soybean. The Gene expression level data set of 9 tissues of soybean were collected from JGI phytozome database to generate heat map. The abscissa shows each organization, vertical coordinate shows each gene. The size and color of circles represent gene expression levels

To obtain more information about the function of G3PDH in soybean, we explored its expression in different organs. The transcriptome data of the *G3PDH* gene in the Phytozome database were used for comprehensive transcriptome analysis of different tissues, including flowers, leaves, nodules, pods, roots, root hairs, seeds, shoot apical meristem (SAM) and stems (Fig. 6).

The results showed that *GmG3PDH9* was only slightly expressed in the flowers, leaves, pods, and seeds, whereas all *GmG3PDHs* were expressed in all tissues. Among them, *GmG3PDH6* was highly expressed in pods, roots, seeds, and SAM; *GmG3PDH8* was highly expressed in seeds; and *GmG3PDH3* was highly expressed in pods and SAM. We speculate that they may be selectively expressed during the growth and development of soybean. Overall, the expression levels of group D genes were significantly higher than those of the other groups, suggesting that group D *G3PDH* genes play fundamental roles in soybean growth and development. Some members of groups C and D had similar expression patterns; therefore, we speculated that their members may have potential functional redundancy or synergy.

### Expression responses of the *G3PDH* gene under virus infection in soybean

**Fig. 7:**
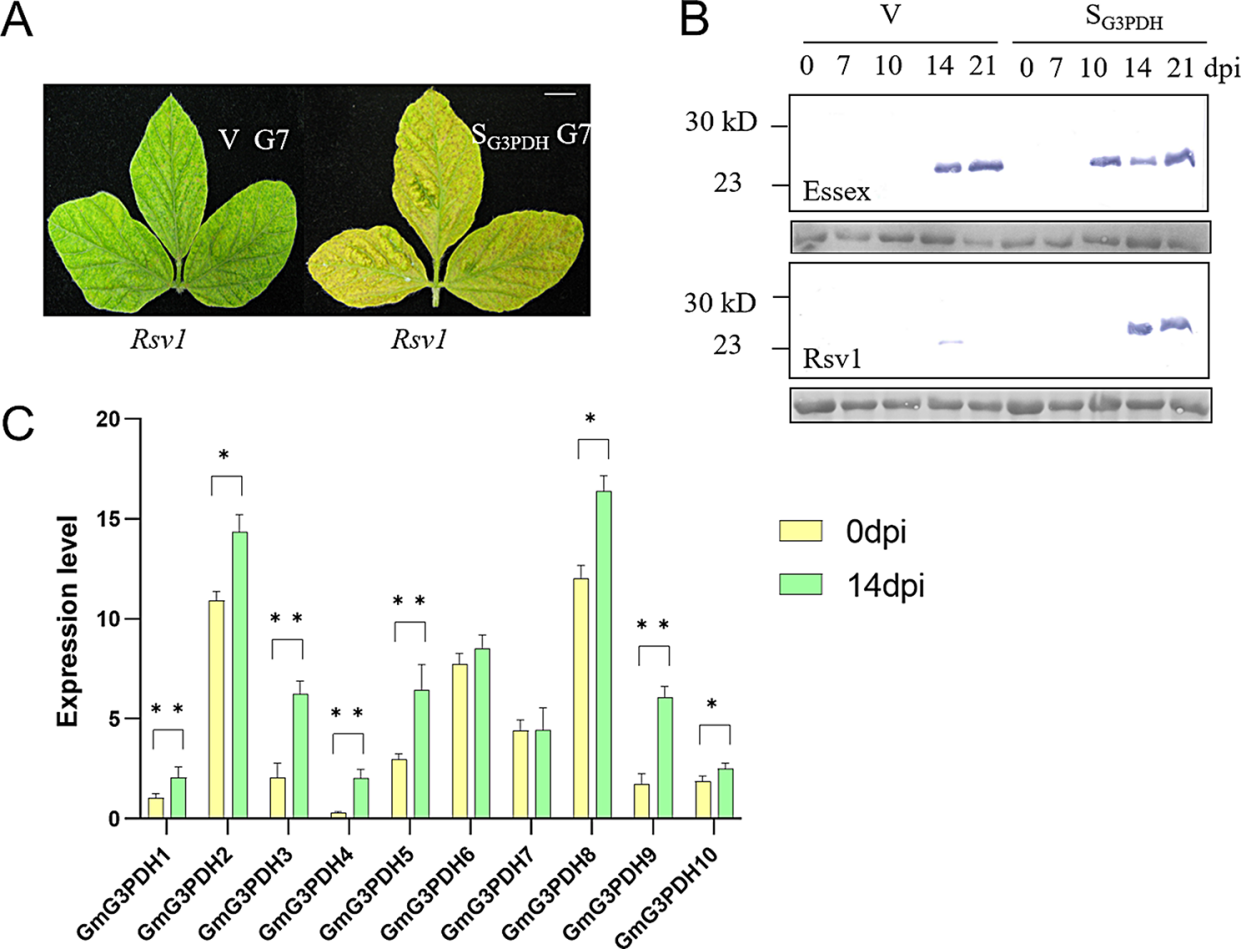
Silencing *GmG3PDH8* alters soybean response to SMV. (**A**) Visual symptoms of Rsv1 plants knocked down for *GmG3PDH8* mRNA expression (S_G3PDH_) or those infected with the control BPMV vector (V) (left panel), which were infected with SMV-G7 on the upper trifoliate leaves. Scale bars = 10 mm. (**B**) Western blot detecting SMV coat protein (CP) in inoculated leaves (0, 7, 10) and systemic leaves (14, 21) of V and S_G3PDH_ plants at indicated days post infection (dpi) with SMV-G7. Proteins were detected using antibodies specific to SMV CP. Total extracts of protein from sample leaves were used. Ponceau staining was used as control for protein loading. (**C**) *GmG3PDHs* gene expression level. Differentially expressed gene data are derived from transcriptomic sequencing, the bar chart was generated using GraphPad Prism 9 software. *Significant difference (*p* < 0.05), **Highly significant difference (*p* < 0.01). Results are representative of 3 independent experiments

In previous studies, we performed transcriptome sequencing on soybean and the results showed that *GmG3PDH8* has the highest expression level which changed significantly after virus inoculation (Fig. 7C). Therefore, we selected *GmG3PDH8* for the validation of disease resistance. BPMV-based vectors are widely used to study gene functions in soybean and common bean [26, 27]. Thus, we used BPMV-VIGS to investigate the roles of the *G3PDH* gene in Rsv1 (SMV-G7 resistant variety) or Essex (Susceptible variety) plants in SMV resistance. We refer to the silencing vector as S_G3PDH_ and the empty BPMV vector (used as a control) as V. We propagated these BPMV-VIGS vectors in susceptible variety Essex plants and inoculated diseased leaves carrying the BPMV recombinant viruses onto the unifoliate leaves of Rsv1 and Essex plants. One week later, we inoculated the trifoliate leaves of Rsv1 and Essex plants with SMV-G7. We examined the local and distal leaves of Rsv1 and Essex plants for SMV accumulation by western blot (Fig. 7B). In *Rsv1* background, the S_G3PDH_ plants showed typical symptoms caused by SMV-G7 infection and accumulated high levels of SMV (when detected by Western blot), while V plants had no obvious symptoms under SMV infection (Fig. 7A). In Essex, the accumulated SMV protein occurred earlier in SG3PDH than in V plants. Therefore, it is likely that G3PDH in the Rsv1 plants mediates SMV resistance.

## Discussion

In previous studies, Ying identified 13 G3PDH members from soybean [28]. Using different identification and screening methods, we identified 10, 6, and 7 *G3PDH* genes in soybean, rice, and alfalfa. To explore the evolutionary process of *G3PDH*, we conducted a phylogenetic analysis of *G3PDH* genes in soybean, rice, and alfalfa and divided them into five groups, named A to E. It is worth noting that may be the evolutionary relationship of group E is older, and all of them are rice *G3PDH* genes; therefore, we speculated that the evolutionary direction of *G3PDH* genes of group E in rice has higher specificity. Group C had the largest gene number and contained all three species; therefore, we speculated that the *G3PDH* genes of Group C had higher environmental adaptability during evolution.

Gene structure analysis of *GmG3PDHs* showed that the intron-exon structure was highly conserved within the same group, except for group C, and had a similar intron region distribution. For example, the coding sequences of group A members were separated by 11 introns, and the coding sequences of group D members were separated by four introns. All family members contain introns, which block the linear expression of genes, indicating that GmG3PDHs may exhibit functional diversity through multiple splicing modifications after transcription.

In addition, the motif distribution of the G3PDH protein was consistent with that of the phylogenetic analysis. Except for group C, the members of the same group had the same motif structure, but the members of different groups were usually different, indicating that the genes in the same group were closely related to the evolutionary process. All family members contain motifs 1 and 6, so we speculate that motifs 1 and 6 were relatively conserved during the evolution of the G3PDH family and play an important role in the function of the G3PDH protein.

In the analysis of cis-acting elements, we found that anaerobic induction response elements had the largest number, and we speculated that soybean requires more anaerobic polypeptides to regulate its metabolic reactions during the growth and development periods. Sachs first reported a sharp decrease in the amount of protein synthesized by the root tips of xerophytes under strict anaerobic conditions [29]. Later studies revealed similar anaerobic polypeptide synthesis patterns in barley, maize, tomato, pea, and soybean [29–35], which confirmed our conclusion. However, the specific regulatory mechanisms underlying soybean growth and development require further investigation.

The results of the expression heat map for different soybean tissues showed that the expression patterns of *GmG3PDHs* were diverse, and different members might show differences in tissue expression. Except for *GmG3PDH9*, the other members were expressed in all organizations. However, their expression levels are quite different; for example, *GmG3PDH6* is highly expressed in pods, roots, seeds, and SAM; *GmG3PDH8* is highly expressed in seeds; and *GmG3PDH3* is highly expressed in pods and SAM. Studies have shown that the expression of soybean disease and insect resistance genes has a synergistic effect [36, 37]. Therefore, we speculated that the function of highly expressed genes in the same tissue may require the synergistic effects of other family members.

Next, we explored the role of *GmG3PDH8* in soybean resistance to SMV using virus induced gene silencing. Western blot results showed that, compared with the control group, the two silencing materials were more susceptible to viral infection, and had more obvious pathological phenotypic characteristics. Therefore, we speculated that GmG3PDH8 plays a positive role in the process of resistance. It is reported that G3PDH regulates the production of G3P, and ulteriorly participates in the process of plant disease resistance through SAR [18–20], which is a key substance in the SAR pathway, and has a positive effect on plant disease resistance [15, 21], plants unable to synthesize G3P have defects in SAR and exogenous G3P complements this defect [12], reduced G3P resulted in decreased AZI1 and DIR1 transcription, both of which are lipid transfer proteins required for SAR [38]. A study had described a root-shoot-root signaling mechanism, which enables plants to exclude non-desirable nitrogen-fixing rhizobia in the root and pathogenic microbes in the shoot simultaneously [39]. There is literature suggesting that the soybean *G3PDH* gene enhances resistance to salt and osmotic stress by regulating the redox state and respiration [40]. Additionally, it may contribute to increased seed oil yield by improving fatty acid composition [41]. Our findings identify GmG3PDH8 as a promising candidate gene for soybean disease resistance. Subsequent functional validation through molecular biotechnology approaches and genetic transformation could facilitate the development of elite soybean cultivars with enhanced SMV resistance.

## Conclusion

In this study, we identified 10 *G3PDH* genes in soybean and conducted a comprehensive analysis of the *G3PDH* gene family. These *GmG3PDHs* were distributed across seven chromosomes and categorized into five groups based on phylogenetic analysis. Through examination of conserved domains, gene structure, cis-acting elements, and the expression patterns of *GmG3PDHs* in various tissues, we observed similarities among *GmG3PDHs* within the same group. Notably, members of group D, including *GmG3PDH6*, *GmG3PDH7*, and *GmG3PDH10* exhibited relatively high expression levels across multiple tissues. Furthermore, we found prominent expression of the GmG3PDH family in pods, roots, seeds, and shoot apical meristems. Silencing of *GmG3PDH8* in response to SMV infection in Essex-Rsv1 strain revealed increased susceptibility to the disease, characterized by earlier virus emergence, higher virus accumulation compared with the control group. This study provides a systematic characterization of the *G3PDH* gene family in soybean, uncovering its critical roles in disease resistance mechanisms. These findings establish a theoretical foundation for developing novel disease-resistant soybean cultivars through targeted genetic engineering or molecular breeding strategies.

## Electronic supplementary material

Below is the link to the electronic supplementary material.

Supplementary Material 1

## Data Availability

All data generated or analyzed during this study is included in this manuscript and is available from the corresponding author upon reasonable request.

## Abbreviations

G3P: Glycerol 3-phosphate
G3PDH: Glycerol 3-phosphate dehydrogenase
VIGS: Virus-Induced Gene Silencing
BPMV: Bean pod mottle virus
SMV: Soybean mosaic virus
GK: Glycerol kinase
DHAP: Dihydroxyacetone phosphate
SAR: Systemic acquired resistance
pIs: Isoelectric points
Chr: Chromosome
AA: Amino Acid
MW: Molecular weight
MeJA: Methyl jasmonate
SAM: Shoot apical meristem
CDS: Coding sequence
UTR: Untranslated regions
CP: Coat protein
