# Supplementary figures and images for: Comprehensive genomic characterization of the soybean G3PDH gene family and its role in virus resistance
Source: BMC Plant Biol. 2025 Sep 1;25:1171. doi: 10.1186/s12870-025-06579-7 (PMC12400704; doi:10.1186/s12870-025-06579-7)

## Slide 1
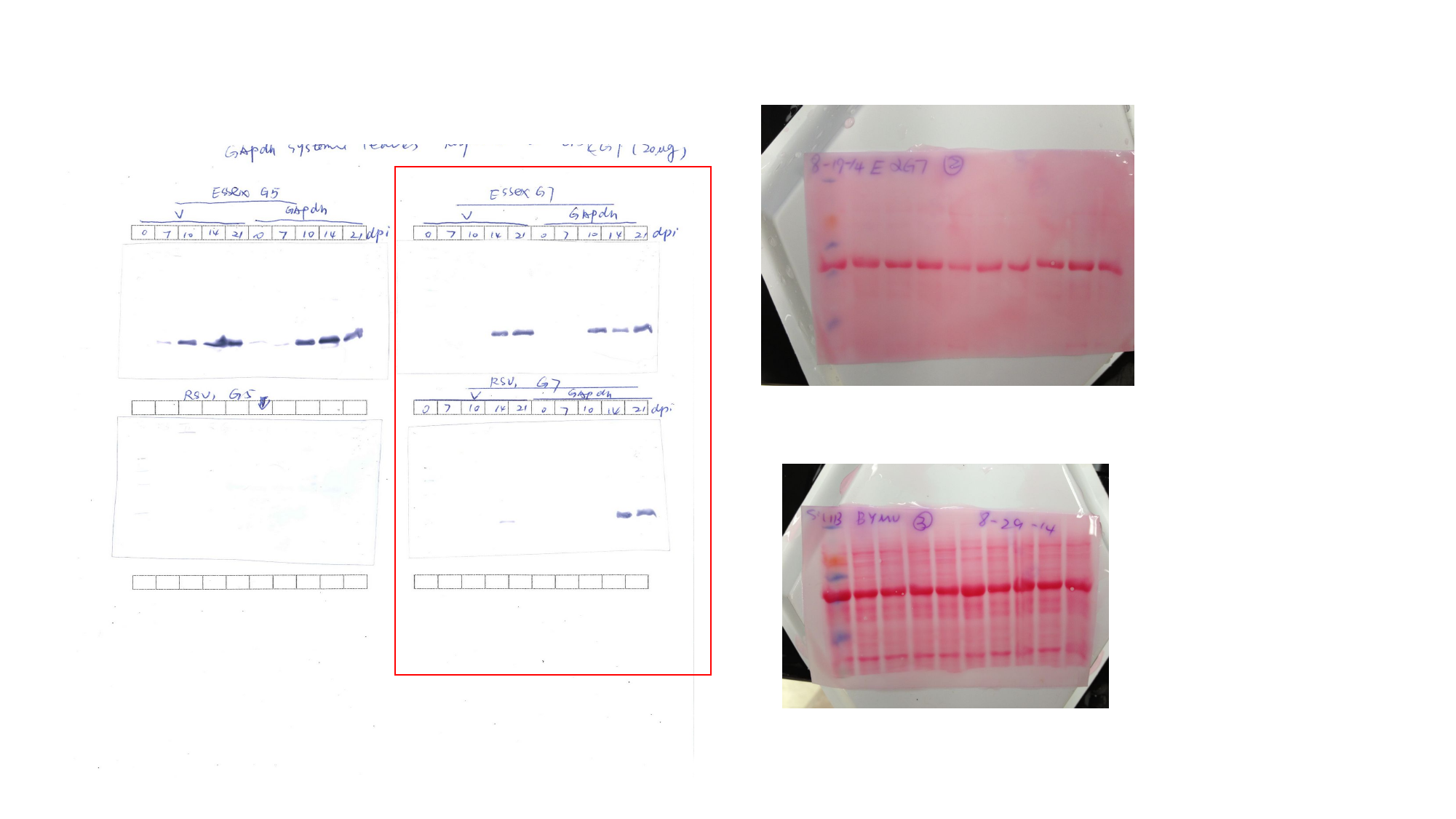

Supplement: Supplementary file 1 — Supplementary Material 1 [file 12870_2025_6579_MOESM1_ESM.pptx]
